# Acute Effect of Toe Cap Choice on Toe Deviation Angle and Perceived Pain in Female Professional Ballet Dancers

**DOI:** 10.1155/2019/9515079

**Published:** 2019-04-10

**Authors:** Annamaria Salzano, Fabiana Camuso, Mario Sepe, Maha Sellami, Luca P. Ardigò, Johnny Padulo

**Affiliations:** ^1^Posturology Center, Salerno 84127, Italy; ^2^College of Arts and Sciences, Sport Science Program, University of Qatar, Doha 2713, Qatar; ^3^Department of Neurosciences, Biomedicine and Movement Sciences, School of Exercise and Sport Science, University of Verona, Verona 37131, Italy; ^4^Department of Psychology, University eCampus, Novedrate 22060, Italy; ^5^Faculty of Kinesiology, University of Split, Split 21000, Croatia; ^6^Sport Performance Lab, Faculty of Kinesiology, University of Split, Split 21000, Croatia; ^7^Tunisian Research Laboratory Sports Performance Optimization, National Center of Medicine and Science in Sport, Tunis 1000, Tunisia

## Abstract

Several classical dance complex movements, such as* pointe*, require body weight to be supported properly to avoid risk of foot injury. Regarding the choice of toe cap for* pointe* shoes, it is unclear which type can better alleviate pain symptoms and toe deviation angle in dancers. The aim of the current crossover study was to investigate the acute effect of using different types of toe caps among well-trained professional dancers on pain perception and toe deviation angle. Ten young female professional dancers volunteered to participate in the study. Each participant was tested during two separate sessions with an interval of 72 h in between. Participants were tested in the two sessions with a standard commercial or a customized prototype toe cap, always with* pointe* shoes, and in randomized order. An anteroposterior X-ray examination was performed separately for each participant and a visual analogue scale for pain perception was administered following each situation (with a standard commercial or a customized prototype toe cap). Significant amelioration was obtained when a customized toe cap prototype was used both for toe deviation angle and for visual analogue scale. Use of a customized toe cap prototype compared to a standard one may acutely reduce both toe deviation angle and pain in elite female professional dancers.

## 1. Introduction

The foot is able to provide the greatest amount of proprioceptive information (PI [1, 2]) and the central processing of this information is considered essential for balance control [3]. For example, knowing how high to jump over an obstacle so as not to strike it with the foot requires high kinaesthetic awareness. As a platform of stability, PI on the foot comes from receptors in the heel front, under the metatarsals' heads, under the big toe, and in the foot's lumbrical muscles [4, 5]. Some authors even claim that some PI comes from the lower limb muscles as well [6]. The plantar aspect of the foot analyses variations in support on the ground and provides all feedback and feedforward information needed for orthostatic equilibrium management [5, 7]. Feedforward involving activation of leg and trunk muscles and feedback involving sensory signals together regulate the maintenance of vertical posture [8–12]. There is a continuous drift of information regarding* status* and function of the musculoskeletal system from PI to the spinal cord,* cerebellum*, and brain; and, when there is a failure in communication process or when one or more sensors send inadequate information, movement efficacy is reduced. As such, standing and moving on a wobbly surface induces body horizontal instability [7].

Alterations in stability or postural control associated with differences in supporting surfaces could influence the responses of available somatosensory information, resulting in increased neuromuscular flow, fatigue, and pain [7, 13]. For example, when standing on one leg or moving on a confined surface, as is the case for dancers (i.e., ballet dancers), this could affect overall postural control [14–16].

Dancers place high demands on the foot and ankle. A dancer's foot is a strong but sensitive, stable, elastic, and specific structure, which supports changes of direction [17]. Several complex movements requiring high-level motor skills and good postural control have been investigated in many studies [18, 19]. Among these actions, dancers are asked to move from bipedal to monopodal support while keeping whole body stability, to jump and turn at the same time, or to perform* passé en demi-pointe* positions. These highly accurate actions in classical ballet require the accurate interpretation of vision and especially of PI coming from the feet [19]. In more complex movements, such as* pointe* exercises, ballerinas have to support their body weight on the tips of fully extended feet within* pointe* shoes sometimes with foot-protecting caps. To perform* pointe* exercises well, it may be useful to make use of targeted exercises, such as* relevés*, which strengthen and give stability to the ankle. This often entails placing a well-trained ballet dancer's body in positions that require high flexibility and controlled power, but even this can put excessive stress on the foot, contributing to the presentation of bunions or metatarsal fractures [20–26].

In* relevé*, simultaneous pain is usually generated or increased with active ankle or foot plantarflexion. During* relevé*, there is extension of a chain of joints starting from the* tibiotalar* one in order to reach the interphalangeal joints. When standing* en pointe*, plantarflexion of the foot and ankle forms a minimum 90° angle [27]. More precisely, during the rise on the tip, the* talus* rotates and slides inside the mortise until it stops, while the* talus-scaphoid*, the* scaphoid-cuneiform-cuboid* (between the metatarsal and* tarsus* bones), and the metatarsal-phalanges provide instep support. The ankle and hindfoot, with help from minor joints, allow for alignment between the knee, the* malleolus*, and toes. Body weight is thus equally distributed and the lower limb's* aplomb* is guaranteed. Muscles responsible for maintaining the tip are toe,* hallux* flexors, and* peronei*. They accomplish the tasks of extending the ankle and joints below it, stabilizing the ankle, and pushing the* talus* forward using the* malleolus* as a* fulcrum*. On the other hand, if this stability is lost due to defects in joint extensions, the dancer will be more prone to injuries, including capsular, ligament, and bone injuries [23].

*Pointe* movement is performed using* pointe* shoes, which help to strengthen the toe/ankle structure and distribute the dancer's weight throughout the foot.* Pointe* shoes help in sufficiently decreasing the load on toes to enable a ballerina to sustain her body weight during vertical positions. However, training with pointe shoes has been demonstrated to increase peak pressure on the foot compared with being barefoot (41* vs.* 86 N/cm^2^ [28]). In addition, moving to a vertical position from a footed one has been shown to increase peak pressure up to 115 N/cm^2^ [29]. A dancer's body is sustained by the toes' tips [30], which can augment pressure on the first toe, forcing it into valgus, thereby possibly contributing to* hallux valgus* deformity [31]. However, it remains unclear whether or not toe cap choice is associated with the greater formation of* hallux valgus* in well-trained dancers. Given the above challenges, together with rigorous performance expectations, investigating and preventing injuries in well-trained ballerinas can be difficult. The aim of the current study was to investigate the effect of using a new CTC (*vs.* an STC) among well-trained professional dancers on pain perception and toe deviation angle (TDA). We hypothesized that both (1) TDA and (2) pain perception were acutely lower with a CTC prototype compared with an STC.

## 2. Materials and Methods

### 2.1. Protocol

The overall design of our study was a crossover study design. Before experimentation started, all participants or, in the case of minors, their parents reviewed and signed consent forms specifically approved by the local ethics committee guidelines. The local ethics committee (Scientific Committee of Posturology Center) approved the entire study design, which was conducted according to the ethical standards of the 1964 Helsinki Declaration.

### 2.2. Subjects

For the sample size calculation, a pilot study that included a sample of 8 participants was performed* a priori* and gave a statistical power greater than 0.81. Therefore, ten young female professional dancers (age: 18.6±3.8 [mean±SD] yrs, body mass: 55.8±6.3 kg, height: 162.9±4.4 cm, training experience 14.4±2.9 yrs, weekly training 10.3±3.9 h) volunteered to participate in this study (Table 1). Participants completed medical history and dietary questionnaires. The inclusion* criteria* were as follows: onset of* hallux valgus* (diagnosed by an orthopedist) and/or sprained first metatarsophalangeal (MTP) joint medial collateral ligaments (sonogram or MRI-verified), being a classical modern female dancer, having training experience in ballet of ≥10 yrs, having an average training frequency of ≥5 h per week, not having been given any chronic medication that could affect inflammation responses or bone development, not being involved in the practice of any structured physical exercise other than ballet or training for it, and not being treated for any muscle or joint injury.

### 2.3. Procedures

**Figure 1:**
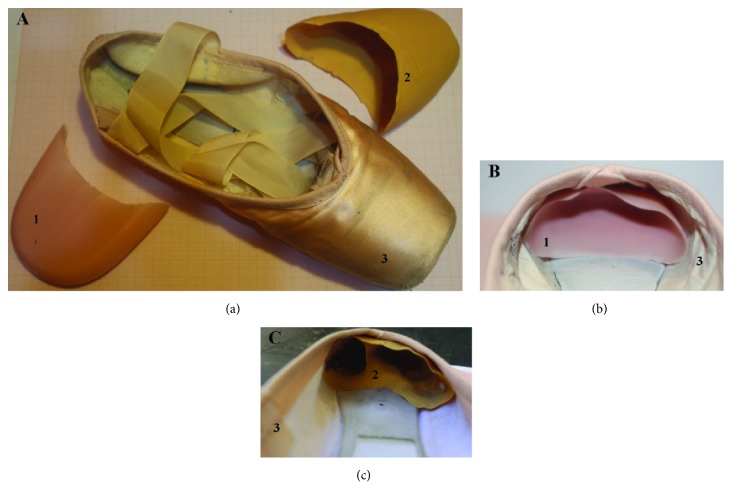
Toe caps: standard commercial toe cap (a), customized toe cap prototype (b), and* pointe* shoe (c).

Each participant was tested during two separate sessions with an interval of 72 h in between. Participants (both of their feet) were tested in the two sessions, once with an STC and once with a CTC, always with* pointe* shoes, and in randomized order (Figure 1). Participants did not know which toe cap condition they were currently being tested in, because they put their shoes on helped by an operator with this operation hidden by a screen.

Participants were also asked to avoid smoking and consuming caffeinated drinks in the 24 h before testing. All procedures were performed at the same daytime period to avoid any circadian effects and at a controlled temperature of 24°C. Participants were requested to refrain from training in the 24 h before testing and to maintain their usual diet. The same investigator always took anthropometric measurements on the morning of the first testing day. Measurements of body mass (kg) and height (cm) were taken for all participants. Body mass was measured to the nearest 0.1 kg, with the subject in light clothing and without shoes, using an electric scale (Model Tanita BC-418, Tokyo, Japan). Each patient also underwent an anteroposterior X-ray examination.

**Figure 2:**
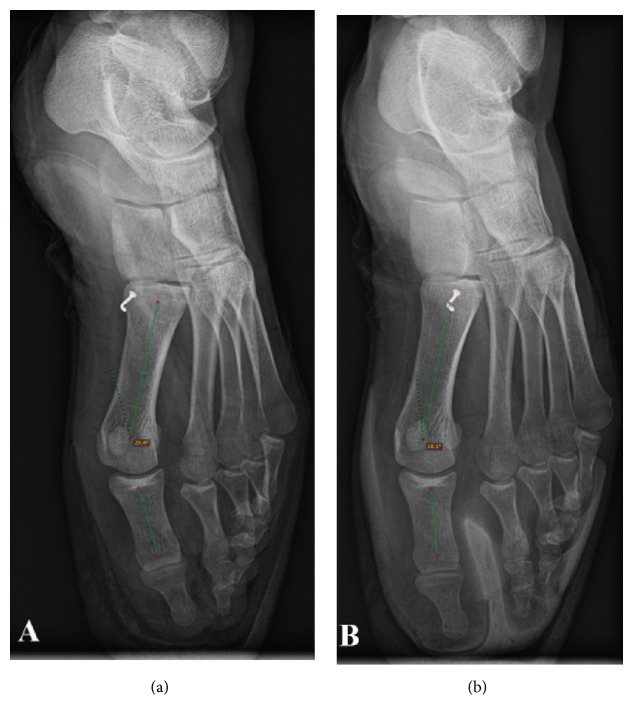
X-ray example: toe deviation angle with standard toe cap (a) and with customized toe cap (b).

Each participant was asked to perform the classical dancer's standard en pointe position for the purposes of radiographic assessment. An X-ray examination was always performed by the same radiologist with 32 years of experience with a FDR D-EVO (Fujifilm, Japan). The position duration was 10 s for both the STC and the CTC. An example is shown in Figure 2. After each position, a VAS for pain perception (a horizontal line, 100 mm long, and with word descriptors at each end) [32] was administered to each participant. Each X-ray was analyzed by the same radiologist with DICOMDIR software (https://www.radiantviewer.com/it) to assess TDA (°), i.e., between the* phalanx* and first metatarsal.

For this investigation, we used silicone gel STCs (Techdance, San Giovanni Teatino, Italy) and custom-built CTCs (E!n Pointe, Salerno, Italy) inside* pointe* shoes (Fouette, Grishko, New York, USA). The STCs were generally rubber (easily deformable) caps. Each CTC (35-shore rigidity) was specifically shaped for each specific dancer's foot by using a handmade biocompatible silicone mix (catalyst+silicon base ratio=1:1). After the silicone mix solidified (catalyzed) around the dancer's foot, the CTC was further milled and its silicon mix excess removed before it was ready for use. The whole manufacturing process took about 30 s [33].

### 2.4. Statistical Analysis

The paired Student's* t*-test was used to investigate whether significant differences existed between the two different conditions (CTC* vs.* STC) in terms of TDA and the VAS. The Shapiro-Francia test was firstly used to assess normal data distribution. Effect size was calculated as Cohen's *d*. For its interpretation, the following thresholds were used: small=0.20, medium=0.50, and large=0.80. The accepted significance level was set at* P*<0.05. The analysis was performed with MedCalc Statistical Software version 18.2.1 (MedCalc, Ostend, Belgium).

## 3. Results

**Figure 3:**
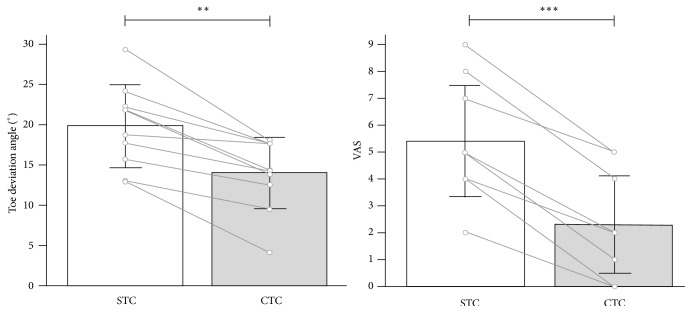
Toe deviation angle with standard toe cap and with customized toe cap for all participants. *∗∗ P*=0.0002, *∗∗∗ P*=0.00005.

The Shapiro-Francia test showed a normal distribution of data with W'=0.971 and* P*=0.891 for TDA and W'=0.999 and* P*≈1.000 for the VAS, respectively. The paired Student's t-test showed that both variables significantly differed between the two conditions. In particular, significant amelioration was obtained when a CTC was used for both TDA, with an average 5.9° reduction (*t*_(1,9)_=-5.854;* P*=0.0002;* d*=1.89, large), and VAS, with an average 3.1 reduction (*t*_(1,9)_=-11.196;* P*=0.00005;* d*=3.66, large). Raw data and analysis outputs are presented in Table 2 and Figure 3, respectively.

## 4. Discussion

The first finding of the current study was that TDA was significantly higher with an STC compared with a CTC prototype. The second finding was that the VAS value for pain perception was significantly higher (i.e., worse) when dancers had shoes with an STC (*P*=0.00005). Any toe cap is strongly embedded inside the* pointe* shoe and tightly encases the toes, so that the ballerina can stand on an oval-shaped support when in the* en pointe* position (Figure 1). A toe cap may be more or less rigid/flexible depending on the model. Traditional models are thin and barely protect the toes' head while providing more support along the foot's sides. According to Teiz et al. [34], force variation in the foot and toe is more dependent on toe length, foot position, and padding or cap use. Force induced on the second toe, the longest one, depends on the toe cap structure and length [34]. According to these authors, during* pointe* or* relevé*, this force increases on the MTP joint. With a toe cap, the distance between the first and second toe reduces allowing for an MTP joint angle deviation reduction (Figure 2).

On the other hand, many studies have investigated different injuries with specific incidences ranging from 27% to 49% in ballet, modern, flamenco, and tap dancers [35–37], with female ballet dancers experiencing the highest incidences. During certain complex movements, such as* pointe*,* relevé*, or* pirouette*, a high force is generated on the dancer's ankle-foot area. During “softer” activities, such a force can reach up to five times the body weight while walking and ~13 times the body weight while running [38]. Considering the great forces developed against the foot and its shape, it is not surprising that toes are the most frequently injured body part and thus the foot represents the main pain site in dance. CTC efficacy in preventing pain is well demonstrated by the current study. This toe cap includes pouches encapsulating the toes inside the unyielding shoe, both cushioning them against shocks to the foot and reducing friction, which potentially causes blistering.

Our study has some limitations. Firstly, it was only focused on the effects resulting from the acute use of customized toe pads exclusively by standard classical dancers in the standing* en pointe* position. It remains unknown what the chronic clinical benefits of the customized toe pads are. Furthermore, sample size was small.

## 5. Conclusions

Toe caps are designed to protect toes and provide grip and stability during complex movements, such as dancing* en pointe*. Whereas some ballerinas still prefer not to use any type of toe cap or pad inside their shoes, others, especially well-trained dancers, are nowadays increasingly more interested in different caps, cushions, and wraps types. In the current study, well-trained professional ballerinas experienced less foot flexibility and deviation angle in addition to less pain perception (almost half) while exercising with a CTC prototype compared with an STC. The effect in terms of injury prevention of CTC use, both chronically and/or during further specific dance movements, is worthy of future investigations.

## Figures and Tables

**Table 1:** Participants' data.

ID		Body mass (kg)	Height (cm)	Age (y)	Training experience in dance (yrs)	Average training frequency (hrs/week)	Dominant foot
Subject 01		50.7	160	15	12	6	Left
Subject 02		62.7	163	17	14	20	Right
Subject 03		61.5	164	25	20	6	Right
Subject 04		49.0	158	22	17	10	Right
Subject 05		67.1	170	19	15	10.5	Right
Subject 06		54.6	160	21	15	10.5	Right
Subject 07		57.8	165	22	16	10.5	Right
Subject 08		52.8	156	16	12	12	Right
Subject 09		54.1	168	16	13	10	Right
Subject 10		47.7	165	13	10	8	Right

*Mean*		*55.8*	*162.9*	*18.6*	*14.4*	*10.3*	
*SD*		*6.3*	*4.4*	*3.8*	*2.9*	*3.9*	
*Range*	*Min*	*47.7*	*156*	*13*	*10*	*6*	
	*Max*	*67.1*	*170*	*25*	*20*	*20*	

*Inclusion criteria*		Onset of *hallux valgus*, classical modern female dancer, training experience in ballet ≥10 years, average training frequency ≥5 hrs/week					
*Exclusion criteria*		Injury (other than *hallux valgus*)					

*Note*

SD=Standard deviation.

**Table 2:** Toe deviation angle and visual analogue scale of each participant.

ID		TDA with STC (°)	TDA with CTC (°)	TDA difference (°)	VAS when dancers are on toes with STC	VAS when dancers are on toes with CTC	VAS difference
Subject 01		29.4	18.1	-11.3	8	4	-4
Subject 02		22.3	17.7	-5.6	5	2	-3
Subject 03		18.8	17.7	-1.1	9	5	-4
Subject 04		24.2	17.7	-6.5	7	5	-2
Subject 05		21.9	13.9	-8.0	4	2	-2
Subject 06		21.9	14.4	-7.5	2	0	-2
Subject 07		17.8	14.3	-3.5	5	2	-3
Subject 08		15.8	12.6	-3.2	5	1	-4
Subject 09		13.1	9.6	-3.5	4	0	-4
Subject 10		13.0	4.2	-8.8	5	2	-3

*Mean*		*19.8*	*14.0*	*-5.9*	*5.4*	*2.3*	*-3.1*
*SD*		*5.1*	*4.4*	*3.1*	*2.1*	*1.8*	*0.9*
*Range*	*Min*	*13.0*	*4.2*	*-1.1*	*2*	*0*	*-2*
	*Max*	*29.4*	*18.1*	*-11.3*	*9*	*5*	*-4*
*P value*		*TDA with STC vs. TDA with CTC P=0.0002*			*VAS with STC vs. VAS with CTC P=0.00005*		

*Note*

TDA=toe deviation angle; STC=standard toe cap; CTC=customized toe cap; VAS=visual analogue scale; SD=standard deviation.

## Data Availability

The data used to support the findings of this study are available from the corresponding author upon request.

## References

[1] van Deursen R. (2008). Footwear for the neuropathic patient: offloading and stability. *Diabetes/Metabolism Research and Reviews*.

[2] Stecco C., Macchi V., Porzionato A. (2010). The ankle retinacula: morphological evidence of the proprioceptive role of the fascial system. *Cells Tissues Organs*.

[3] Han J., Waddington G., Adams R., Anson J., Liu Y. (2015). Assessing proprioception: a critical review of methods. *Journal of Sport and Health Science*.

[4] Fitz-Ritson D. (1982). The anatomy and physiology of the muscle spindle and its role in posture and movement: a review. *The Journal of the Canadian Chiropractic Association*.

[5] Robbins S., Waked E., McClaran J. (1995). Proprioception and stability: foot position awareness as a function of age and footware. *Age and Ageing*.

[6] Padulo J., Powell D., Milia R., Ardigò L. P. (2013). A Paradigm of Uphill Running. *PLoS ONE*.

[7] Mohapatra S., Kukkar K. K., Aruin A. S. (2014). Support surface related changes in feedforward and feedback control of standing posture. *Journal of Electromyography & Kinesiology*.

[8] Alexandrov A., Frolov A., Horak F., Carlson-Kuhta P., Park S. (2005). Feedback equilibrium control during human standing. *Biological Cybernetics*.

[9] Belen'kii V. E., Gurfinkel' V. S., Pal'tsev E. I. (1967). Control elements of voluntary movements. *Biofizika*.

[10] Horak F. B., MacPherson J. M., Peterson B. W. (1996). *Postural Orientation and Equilibrium*.

[11] Massion J. (1992). Movement, posture and equilibrium: interaction and coordination. *Progress in Neurobiology*.

[12] Park S., Horak F. B., Kuo A. D. (2004). Postural feedback responses scale with biomechanical constraints in human standing. *Experimental Brain Research*.

[13] Gatev P., Thomas S., Kepple T., Hallett M. (1999). Feedforward ankle strategy of balance during quiet stance in adults. *The Journal of Physiology*.

[14] Leanderson J., Eriksson E., Nilsson C., Wykman A. (1996). Proprioception in classical ballet dancers: A prospective study of the influence of an ankle sprain on proprioception in the ankle joint. *The American Journal of Sports Medicine*.

[15] Schmit J. M., Regis D. I., Riley M. A. (2005). Dynamic patterns of postural sway in ballet dancers and track athletes. *Experimental Brain Research*.

[16] Simmons R. W. (2009). Sensory organization determinants of postural stability in trained ballet dancers. *International Journal of Neuroscience*.

[17] Ahonen J. (2008). Biomechanics of the foot in dance: a literature review. *Journal of Dance Medicine & Science*.

[18] Costa M. S. S., Ferreira A. S., Felicio L. R. (2013). Static and dynamic balance in ballet dancers: a literature review. *Fisioterapia e Pesquisa*.

[19] de Mello M. C., de Sá Ferreira A., Ramiro Felicio L. (2017). Postural control during different unipodal positions in professional ballet dancers. *Journal of Dance Medicine & Science*.

[20] Brown T. D., Micheli L. J. (2004). Foot and ankle injuries in dance. *American journal of orthopedics (Belle Mead, N.J.)*.

[21] Harrington T., Crichton K. J., Anderson I. F. (2016). Overuse ballet injury of the base of the second metatarsal. *The American Journal of Sports Medicine*.

[22] Kadel N. J. (2006). foot and ankle injuries in dance. *Physical Medicine and Rehabilitation Clinics of North America*.

[23] Lin C., Su F., Wu H. (2007). Ankle biomechanics of ballet Dancers in relevé en pointé dance. *Research in Sports Medicine*.

[24] Macintyre J., Joy E. (2000). Foot and ankle injuries in dance. *Clinics in Sports Medicine*.

[25] Micheli L. J., Sohn R. S., Solomon R. (1985). Stress fractures of the second metatarsal involving Lisfranc's joint in ballet dancers. A new overuse injury of the foot. *The Journal of Bone and Joint Surgery*.

[26] Prisk V. R., O'Loughlin P. F., Kennedy J. G. (2008). Forefoot injuries in dancers. *Clinics in Sports Medicine*.

[27] Shah S. (2009). Determining a young dancers readiness for dancing on pointe. *Current Sports Medicine Reports*.

[28] Barringer J., Schlesinger S. (1991). *The Pointe Book*.

[29] Albers D., Hu R., McPoil T. G., Cornwall M. W. (1993). Comparison of foot plantar pressures during walking and en pointe. *Kinesiology and Medicine for Dance*.

[30] Kadel N., Boenisch M., Teitz C., Trepman E. (2005). Stability of Lisfranc joints in ballet pointe position. *Foot & Ankle International*.

[31] Tuckman A. S., Werner F. W., Bayley J. C. (1991). Analysis of the forefoot on pointe in the ballet dancer. *Foot & Ankle*.

[32] Crichton N. (2001). Visual analogue scale (VAS). *Journal of Clinical Nursing*.

[33] Russo L., Bartolucci P., Ardigò L. P., Padulo J., Pausic J., Dello Iacono A. (2018). An exploratory study on the acute effects of proprioceptive exercise and/or neuromuscular taping on balance performance. *Asian Journal of Sports Medicine*.

[34] Teitz C. C., Harrington R. M., Wiley H. (1985). Pressures on the foot in pointe shoes. *Foot & Ankle International*.

[35] Jacobs C. L., Hincapié C. A., Cassidy J. D. (2012). Musculoskeletal injuries and pain in dancers: a systematic review update. *Journal of Dance Medicine & Science*.

[36] Mayers L., Judelson D., Bronner S. (2003). The prevalence of injury among tap dancers. *Journal of Dance Medicine Science*.

[37] Shah S., Weiss D. S., Burchette R. J. (2012). Injuries in professional modern dancers: incidence, risk factors, and management. *Journal of Dance Medicine & Science*.

[38] MacLean C. L., Davis I. S., Hamill J. (2009). Influence of running shoe midsole composition and custom foot orthotic intervention on lower extremity dynamics during running. *Journal of Applied Biomechanics*.

